# Targeting host metabolism by inhibition of acetyl-Coenzyme A carboxylase reduces flavivirus infection in mouse models

**DOI:** 10.1080/22221751.2019.1604084

**Published:** 2019-04-19

**Authors:** Nereida Jiménez de Oya, William P. Esler, Kim Huard, Ayman F. El-Kattan, Georgios Karamanlidis, Ana-Belén Blázquez, Priscila Ramos-Ibeas, Estela Escribano-Romero, Andrés Louloudes-Lázaro, Josefina Casas, Francisco Sobrino, Kyle Hoehn, David E. James, Alfonso Gutiérrez-Adán, Juan-Carlos Saiz, Miguel A. Martín-Acebes

**Affiliations:** a Department of Biotechnology, Instituto Nacional de Investigación y Tecnología Agraria y Alimentaria (INIA), Madrid, Spain; b Worldwide Research and Development Pfizer, Cambridge, MA, USA; c Department of Animal Reproduction, INIA, Madrid, Spain; d Department of Biomedicinal Chemistry, Institute for Advanced Chemistry of Catalonia (IQAC-CSIC) and CIBEREHD, Barcelona, Spain; e Department of Virology and Microbiology, Centro de Biología Molecular “Severo Ochoa” (CSIC-UAM), Madrid, Spain; f School of Biotechnology and Biomolecular Sciences, University of New South Wales, Sydney, Australia; g Charles Perkins Centre, School of Life and Environmental Sciences, Sydney Medical School, University of Sydney, Australia; * Present address: Cardiometabolic Disorders Amgen Discovery Research, Thousand Oaks, California91320, USA.

**Keywords:** Flavivirus, West Nile virus, dengue virus, Zika virus, antivirals

## Abstract

Flaviviruses are (re)-emerging RNA viruses strictly dependent on lipid metabolism for infection. In the search for host targeting antivirals, we explored the effect of pharmacological modulation of fatty acid metabolism during flavivirus infection. Considering the central role of acetyl-Coenzyme A carboxylase (ACC) on fatty acid metabolism, we analyzed the effect of three small-molecule ACC inhibitors (PF-05175157, PF-05206574, and PF-06256254) on the infection of medically relevant flaviviruses, namely West Nile virus (WNV), dengue virus, and Zika virus. Treatment with these compounds inhibited the multiplication of the three viruses in cultured cells. PF-05175157 induced a reduction of the viral load in serum and kidney in WNV-infected mice, unveiling its therapeutic potential for the treatment of chronic kidney disease associated with persistent WNV infection. This study constitutes a proof of concept of the reliability of ACC inhibitors to become viable antiviral candidates. These results support the repositioning of metabolic inhibitors as broad-spectrum antivirals.

## Introduction

The genus *Flavivirus* includes 53 closely related species of positive-strand RNA viruses transmitted by vectors (mosquitoes and ticks) [1]. Flaviviruses are responsible for mild-flu like symptoms, neurological syndromes, birth defects, and hemorrhagic fevers. For instance, West Nile virus (WNV) provokes outbreaks of febrile illness, encephalitis, acute flaccid-paralysis and can induce long lasting sequelae and a chronic renal disease associated with persistent infection, dengue virus (DENV) causes about 100 million cases of disease each year including hemorrhagic fevers, and Zika virus (ZIKV) is responsible for birth defects (microcephaly) and neurological syndromes [2–5]. The increase in worldwide travel and trade, global warming, and urbanization are facilitating the colonization of new territories by pathogenic flaviviruses, putting human and animal health at risk. This can be easily exemplified by the emergence of WNV and ZIKV in the Americas from 1999 and 2015, respectively. Despite their clinical relevance, most flaviviruses are still neglected pathogens and there are no specific licensed therapies to combat them. Consequently, there is an urgent need for effective therapies not only against recognized pathogenic flaviviruses, but also potentially suitable against future flaviviral threats.

The advances in the understanding of virus-host interactions have led to the identification of the essential cellular pathways for infection, which has allowed new antiviral approaches directed against host factors. In comparison to direct-acting antivirals that target unique viral factors, host-directed antivirals should be advantageous due to their potential broad spectrum and their theoretical higher genetic barrier to the selection of resistant mutants [6]. Flaviviruses ideally constitute an appropriate objective for this kind of host-directed antiviral discovery that may result in the identification of pan-flaviviral drugs, providing low cost but effective control tools [7–9].

Most viruses, including flaviviruses, are forced to co-opt for specific cellular lipids to complete their life cycles. Due to this fact, lipid metabolism has become an attractive target for host-directed antiviral interventions [10,11]. For instance, a wide variety of viruses are strictly dependent on fatty acid metabolism [12–17]. Flaviviruses also share this dependence on fatty acid metabolism for infection. Fatty acids provide the building blocks for the synthesis of complex lipids that are necessary for flavivirus replication and particle morphogenesis, promote energy production in infected cells, and participate in the immune response [18–23]. A key step within fatty acid metabolism, catalyzed by the acetyl-CoA carboxylase (ACC), is the ATP dependent carboxylation of acetyl-CoA to produce malonyl-CoA [24]. The malonyl-CoA is the essential and rate-limiting substrate for *de novo* lipogenesis and inhibits the transport of long chain fatty acyl-CoAs across the mitochondrial membrane where they can enter fatty acid oxidation. Therefore, ACC regulates both fatty acid synthesis and oxidation. The central role of ACC in fatty acid metabolism allows this enzyme to be a potential target for the treatment of metabolic diseases and cancer [25]. Despite the strong evidence supporting the role of fatty acid metabolism during viral infections, to our knowledge, the potential of ACC as an antiviral target has not been evaluated *in vivo*.

In this study, we analyzed the antiviral effect resulting from modulation of fatty acid metabolism through ACC inhibition. Pharmacological blockage of ACC inhibited flavivirus multiplication in cultured cells and reduced viral load in mice. Our results provide experimental evidence to support the hypothesis that ACC is as a druggable target suitable for antiviral intervention.

## Materials and methods

### Ethics statement

Mouse pharmacokinetic studies were conducted in AAALAC accredited facilities and reviewed and approved by Pfizer Institutional Animal care and Use Committee. All experiments with infectious viruses were conducted in biosafety level 3 facilities and approved by the Ethical Committee of Animal Experimentation of Instituto Nacional de Investigación y Tecnología Agraria y Alimentaria (INIA, Madrid, Spain) and by the Division of Animal Protection of the Comunidad de Madrid (PROEX 187/17). Animals were handled in strict accordance with the guidelines of the European Community 86/609/CEE.

### Infections

Infectious virus manipulations were performed in biosafety level 3 facilities. The origin of WNV NY99, ZIKV PA259459 and DENV-2 has been described [26]. Infections and virus titrations were carried as described [26] using Vero cells (catalog no. CCL-81; ATTC, Manassas, VA). Unless otherwise specified, Vero cells grown on 24-well culture plates (∼2 × 10^5^ cells/well) were infected with the viruses at a multiplicity of infection (MOI) of 1 plaque forming units (PFU)/cell. Unless otherwise specified, inhibitors were added to cultured cells 1 h after virus inoculation when viral inoculum was replaced by culture medium supplemented with 1% fetal bovine serum. At the end of the treatment (24 h of infection for WNV and ZIKV and 48 h for DENV) virus released to the supernatant of infected cultures was harvested and subsequently titrated. Virus titer was calculated as the number of (PFU)/mL by standard plaque assay using semisolid agarose medium. Antiviral potencies were estimated by calculating the absolute half maximal effective concentration [EC_50_] from a 4 point dose–response curve including 0, 0.5, 1 and 5 µg/mL of each drug. When the smallest concentration of the compound tested induced a reduction higher than 50%, it was displayed that the EC_50_ was lower than this concentration. Virucidal assays were performed as described [34]. Briefly, the same amount of WNV (∼ 3 × 10^8^ PFU) was preincubated with the compounds (5 µg/mL) or drug vehicle for 1 h at 37°C in culture medium and then titrated by standard plaque assay to determine the remaining infectivity. For time of addition assays, Vero cells were infected with WNV (MOI of 1 PFU/cell) and treated with the compounds (5 µg/mL) or the equivalent drug vehicle at the desired time points: only 1 h before virus inoculation (drug pre-infection), 1 h after virus inoculation (drug post-infection), 1 h before virus inoculation and during infection (drug pre-infection and post-infection), or left untreated (vehicle). Virus yield in supernatant was determined by plaque assay 24 h after infection.

### Inhibitors

(-)-Epicgallocathechin gallate, EGCG (catalog no. 0981 S) was from Extrasynthese (Genay, France). The procedures for the synthesis of PF-05175157 [27], PF-05206574 [28] and PF-06256254 [29] have been previously reported. PF-05175157, PF-05206574 and PF-06256254 were synthesized by Pfizer according to described procedures [27–29]. PF-05175157 is commercially available from Sigma (catalog no. PZ0299). Apparent permeability was assessed in low-efflux MDCKII cells [30]. *In vitro* potencies of the inhibitors were determined using a radiometric assay [27]. For tissue culture experiments, drugs were suspended in DMSO. Control cells were treated in parallel with the same amount of DMSO (vehicle). Cell viability was estimated in uninfected cells by ATP measurement using Cell Titer Glo luminescent cell viability assay (catalog no. G7579; Promega, Madison, WI). For this purpose, cells were seeded in 96-well plates and were treated with the inhibitors in culture medium supplemented with 1% fetal bovine serum for 24 or 48 h. Cells were lysed by adding equal amounts of Cell Titer Glo reagent (100 µL/well) and gentle rocking (2 min) in an orbital shaker. The amount of ATP in each sample was determined by luminescence measurement using white microplates and a TECAN Genius (Zurich, Switzerland) microplate reader.

### Mice

Pharmacokinetics was analyzed after a single oral (p.o.) dose of the compounds (in a 0.5% methyl cellulose suspension) administered to fasted male mice. A dose of 15 mg/kg for PF-05175157 or 100 mg/kg in the case of PF-05206574 and PF-06256254 was analyzed. Antiviral activity *in vivo* was determined using eight-week-old *Swiss* albino CD-1 female mice (Envigo, Huntingdon, UK). Animals were treated with PF-05175157 (20 mg/kg) suspended in 1% carboxymethylcellulose by oral gavage (p.o.) twice a day from 1 d before infection with WNV (1 × 10^4^ PFU/mouse intraperitoneally, i.p.) and up to 7 days post-infection. Control mice were treated in parallel with drug vehicle (carboxymethylcellulose). For experiments evaluating the effect of genetic deletion of *ACC2* gene on WNV infection, a breeding colony of C57BL/6 *ACC2*^-/-^ mice was established from two heterozygous Acab^tm1Dejs^ females [31]. Age- and sex-matched eight-week-old *ACC2*^-/-^ and control wild type (WT) mice were challenged with WNV (1 × 10^4^ PFU/animal, i.p.). Animals were monitored daily and received water and food *ad libitum.* Animals exhibiting clear signs of disease were anesthetized and sacrificed as were all surviving mice at the end of the experiment.

### Lipidomics

Lipid extractions, identification, and quantification by mass spectrometry of Vero cells (10^6^ cells/determination) treated with the inhibitors (5 µg/mL, 24 h) or drug vehicle were performed as described [32].

### Virus-like particle (VLP) assay

The amount of VLP released by HeLa3-WNV cells was determined by enzyme-linked immunodot assay [32] using mouse monoclonal anti-WNV envelope protein antibody (catalog no. MAB8150; EMD Millipore, Billerica, MA). To this end, HeLa3-WNV were treated with drug vehicle or ACC inhibitors (5 µg/mL) for 20 h. At this time, culture medium was removed and replaced by fresh medium containing the ACC inhibitors or drug vehicle, and cells were further incubated 4 h more. Culture supernatants containing the VLPs secreted during this 4 h of incubation were then harvested and the amount of VLPs was determined by enzyme-linked immunodot assay.

### Immunofluorescence

Immunofluorescence detection of dsRNA using monoclonal antibody J2 (Catalog no. 10010200; Scicons, Szirák, Hungary) in cells infected with WNV (MOI of 1 PFU/cell) and treated with 5 µg/mL of each ACC inhibitor, or drug vehicle, was performed as described [20]. Samples were observed with a confocal laser scanning microscope Leica SPE (Leica, Wetzlar, Germany).

### Quantification of viral RNA

RNA was automatically extracted using a QIAcube equipment (QIAGEN GmbH, Hilden, Germany) and QIAamp viral RNA Mini Kit (QIAGEN, catalog no. 52906) for sera or RNeasy Mini Kit (QIAGEN, catalog no. 74106) for tissue homogenates. The content of viral RNA in each sample was analyzed by real-time fluorogenic reverse transcriptase PCR by comparison with previously titrated samples and determined as PFU equivalents/mL [22]. In the case of experiments determining the effect of ACC inhibitors on WNV multiplication in Vero cells, these were initially infected with a MOI of 1 PFU/cell and treated with 5 µg/mL of each ACC inhibitor, or drug vehicle, for 24 h.

### Statistical analysis

Statistical analyses were performed using GraphPad PRISM 7 for Windows (GraphPad Software Inc., San Diego, CA). Virus yield were compared using Analysis of the Variance (ANOVA) and *t*-test applying Bonferroni’s correction for multiple comparisons. Tissue and serum viral loads were analyzed by Mann–Whitney test. Lipidomic data were analyzed using multiple *t*-test and Sidak-Bonferroni correction for multiple comparisons. Statistically significant differences are indicated in the figures: *P < 0.05, **P < 0.01, ***P < 0.001. The number of biological replicates or animals analyzed in each group is denoted by *n* in the figure legends. Unless otherwise specified data are presented as the mean ± standard deviation (SD).

## Results

### Antiviral activity of ACC inhibitors against medically relevant flaviviruses

**Figure 1.**
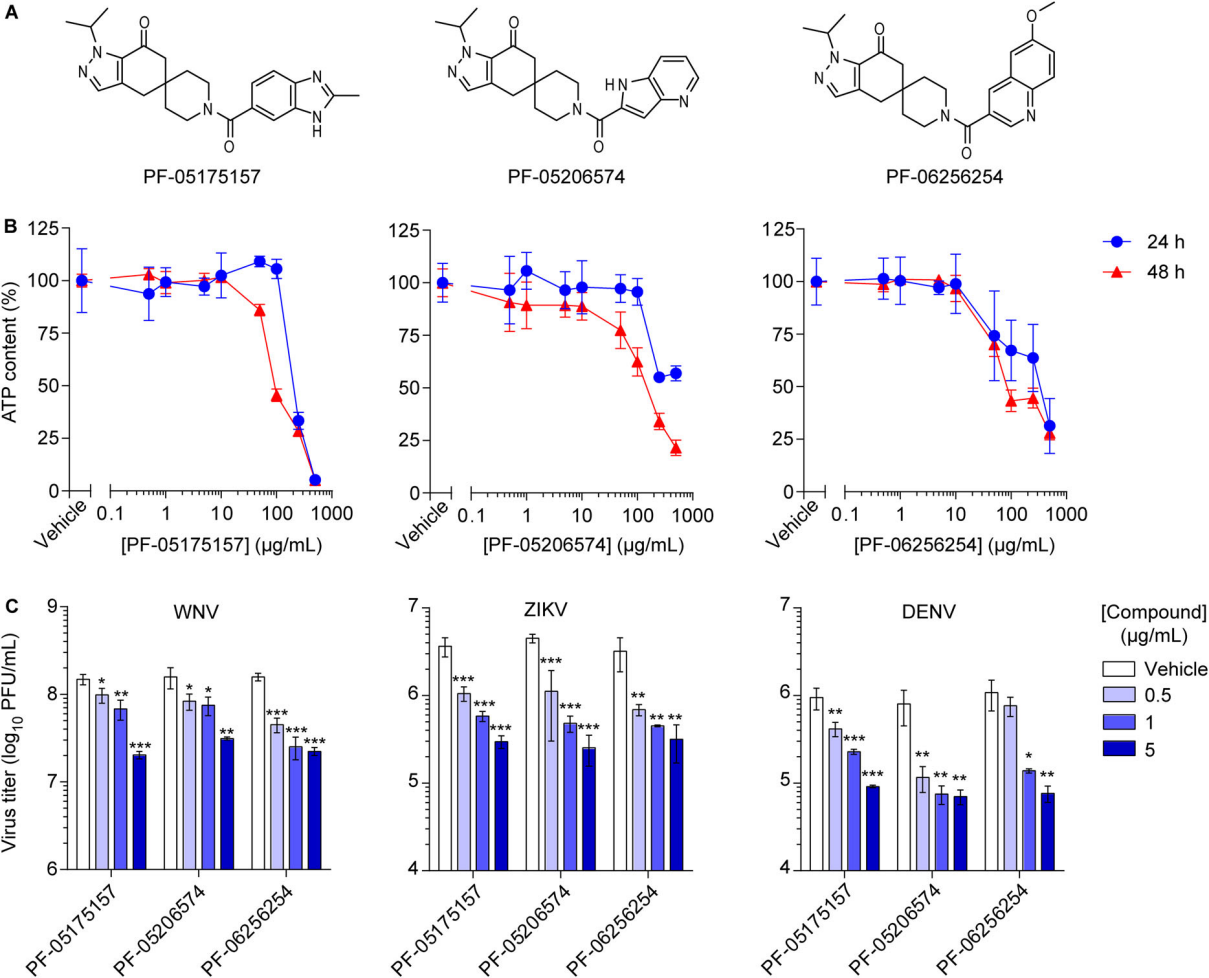
**ACC inhibitors reduce flavivirus multiplication at non-cytotoxic concentrations. (A)** Chemical structure of ACC inhibitors PF-05175157, PF-05206574 and PF-06256254. **(B)** Effect of ACC inhibitors on cell viability. Vero cells were treated for 24 or 48 h with PF-05175157, PF-05206574 or PF-06256254 and cell viability was determined by measuring cell ATP concentrations (*n *= 4). **(C)** Inhibition of flavivirus multiplication by ACC inhibitors. Vero cells were infected with the flaviviruses WNV, ZIKV or DENV (MOI of 1 PFU/cell) and 1 h after virus addition, viral inoculum was removed and culture medium containing the various concentrations of the compounds was added. The virus yield was determined as the viral titer in infected supernatants after 24 h (WNV and ZIKV) or 48 h (DENV) of infection (*n* = 3). Two tailed Student’s *t*-test *P* values between Vehicle and each compound concentration are Bonferroni-corrected for multiple comparisons. *P < 0.05, **P < 0.01, ***P < 0.001. Data represent the means ± SDs.

A series of spirocyclic ketone-containing ACC inhibitors was selected, namely PF-05175157 [27], PF-05206574 [28], and PF-06256254 [29] (Figure 1A). All three compounds exhibited good potencies against the two isoforms of the enzyme (ACC1 and ACC2) from human and rat (half-maximal inhibitory concentration [IC_50_] = 10–27 nM). The apparent permeability of the compounds (*P*_ap*p*_ = 7 × 10^6^–24 × 10^6^ cm/s) supported a high exposure in cellular systems (Table 1). The cytotoxicity of the compounds was evaluated by cellular ATP determination (Figure 1B). A dose-dependent reduction of cell viability was observed at high drug concentrations after 24 and 48 h of treatment (half-maximal cytotoxic concentration [CC_50_] > 500 µM and > 200 µM respectively) (Table 1). Given the good potency, high exposure and low cytotoxicity, the antiviral effect of the ACC inhibitors on the infection of three medically relevant flaviviruses (WNV, ZIKV, and DENV) was assayed (Figure 1C). Based on the different growth kinetics of the viruses, the antiviral activity was assessed after 24 h of infection for WNV and ZIKV and after 48 h of infection for DENV [26]. The three compounds significantly reduced the virus yield in a dose-dependent manner (Figure 1C) and exhibited similar antiviral potencies (half maximal effective concentration [EC_50_] in the low µM range) (Table 1). To evaluate the balance between efficacy and safety of the drugs, the relation between cytotoxicity and antiviral activity was assessed by calculating the selectivity index (SI) of each compound against WNV, ZIKV, and DENV. In all cases, SI evidenced a good relation between efficacy and safety of the drugs (SI > 100) although some variation was observed among viruses and compounds (Table 1). Overall, these results suggest the broad spectrum antiviral effect of the ACC inhibitors and support that this series of compounds could be good lead candidates for additional development.

**Table 1.** Chemical properties and antiviral activities of ACC inhibitors

		PF-05175157	PF-05206574	PF-06256254
MW^a^		405.49	391.47	432.51
*P*_app_ (cm/s)^b^		7 × 10^−6^	19 × 10^−6^	24 × 10^−6^
IC_50_ (nM)^c^	human ACC1	27 ± 3	19 ± 4	10 ± 2
	human ACC2	33 ± 4	22 ± 9	10 ± 2
	rat ACC1	24 ± 1	14 ± 3	11
	rat ACC2	50 ± 3	18 ± 4	15
CC_50_ (µM)^d^	24 h	590 ± 37	>1277	718 ± 314
	48 h	236 ± 11	415 ± 67	202 ± 22
EC_50_ (µM)^e^	WNV	2.7 ± 1.3	1.9 ± 0.9	<1.2
	ZIKV	<1.2	<1.3	<1.2
	DENV	1.0 ± 0.3	<1.3	1.5 ± 0.4
SI (CC_50_/EC_50_)^f^	WNV	219	>672	>598
	ZIKV	>492	>982	>598
	DENV	236	>319	135

^a^MW, molecular weight.

^b^*P*_app_, apparent permeability.

^c^IC_50_, inhibitory concentration. *In vitro* potencies were determined using a radiometric assay and are reported as the geometric mean ± the standard error of the mean (*n* = 3; in the case of PF-06256254 and rat ACC1 and ACC2 *n* = 1).

^d^CC_50_, cellular cytotoxicity. Concentration of the compound that resulted in 50% reduction of ATP content (*n* = 4). Data are expressed as means ± SD. The value >1277 indicated that less than 50% inhibition was observed at 1277 µM (500 µg/mL), the highest concentration that was tested.

^e^EC_50_, effective concentration. Concentration of the compound at which virus replication was inhibited by 50%. Antiviral activities were evaluated in Vero cells (MOI 1 PFU/cell) at 24 h for WNV and ZIKV and 48 h for DENV (*n* = 3). The value <1.2 or <1.3 indicates that inhibition higher than 50% was observed at the lowest concentration of the compound tested (0.5 µg/mL; 1.2 µM for PF-05175157 and PF-06256254 or 1.3 µM for PF-05206574). Data are expressed as means ± SD.

^f^SI, selectivity or therapeutic index (CC_50_/EC_50_) were calculated at 24 h for WNV and ZIKV and 48 h for DENV.

### Preclinical pharmacokinetics of the ACC inhibitors

**Figure 2.**
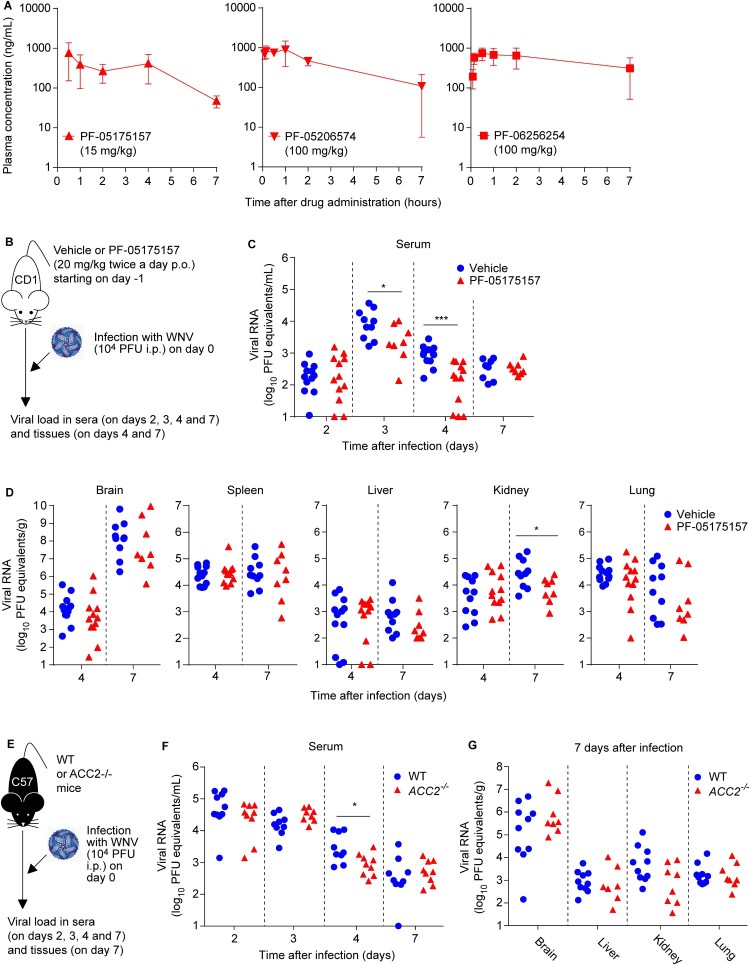
**Inhibition of ACC reduces viral load in mice. (A)** Total plasma level curves of the compounds after a single oral dose (p.o.) of 15 mg/kg for PF-05175157 or 100 mg/kg in the case of PF-05206574 and PF-06256254. Compounds were administered to fasted male mice by oral gavage (*n* = 3). Data represent the means ± SDs. **(B-D)** Antiviral activity of PF-05175157 against WNV in mice. **(B)** Experimental design. Eight-week-old female CD-1 mice were treated with 20 mg/kg of PF-05175157 twice a day p.o. starting 1 d before infection. Animals were infected by the i.p. route with 1 × 10^4^ PFU/mouse of WNV. **(C** and **D)** Viral burden in (C) serum and (D) tissues (brain, spleen, liver, kidney and lungs) was determined by quantitative RT-PCR at the indicated times post-infection. Symbols represent individual mice pooled from two independent experiments (*n* = 8–12). Two-tailed Mann Whitney test *P* values are indicated in (B) *P = 0.0206, ***P < 0.0001 and (C) *P = 0.0266. **(E-G)** Infection with WNV in ACC2-deficient mice. **(E)** Experimental design. Eight-week-old sex-matched WT and *ACC2*^-/-^ mice were infected by the i.p. route with 1 × 10^4^ PFU/mouse of WNV (*n* = 10 for WT; *n* = 9 for *ACC2*^-/-^). Viral load in serum **(F)** or organs (brain, liver, kidney and lung) **(G)** of infected WT or *ACC2*^-/-^ mice was determined by quantitative RT-PCR at the indicated times post-infection (*n* = 9–10 for WT; *n* = 8–9 for *ACC2*^-/-^). Symbols represent individual mice. Two-tailed Mann Whitney test *P* values is indicated in (E) *P = 0.0244.

Considering that the mouse model is commonly used for flavivirus research, the pharmacokinetic of the ACC inhibitors was assessed in this species. The mean plasma concentration-time curves after oral administration (p.o.) of the inhibitors are illustrated in Figure 2A and the preclinical pharmacokinetics properties are summarized in Table 2. Following oral administration, the compounds were rapidly absorbed (time to reach the maximum plasma-concentration [*T_max_*] = 0.383–1.67 h). All three compounds displayed good oral systemic exposure (area under the plasma-concentration curve [AUC*_Exp_*]* *= 2170–3700 ng×h/mL). Compared to PF-05175157, a higher dose was required for PF-05206574 and PF-06256254 to achieve total plasma concentrations (maximum-plasma concentration [*C_max_*] = 760–1070 ng/mL) that were within the required range for the antiviral activity (Figure 1 B and Table 1). Accordingly, the pharmacokinetics of the compounds supported their potential antiviral activity *in vivo*.

**Table 2.** Preclinical pharmacokinetics of the ACC inhibitors.

	PF-05175157	PF-05206574	PF-06256254
Dose (mg/kg)^a^	15	100	100
AUC_(Exp)_ (ng × h/mL)^b^	2170 ± 384	3700 ± 1440	2900 ± 1070
*C_max_* (ng/mL)^c^	903 ± 495	760 ± 240	1070 ± 452
*T_max_* (h)^d^	1.67 ± 2.02	0.383 ± 0.20	0.550 ± 0.43

^a^Route of administration p.o. (oral gavage), using crystalline material as a 0.5% methyl cellulose suspension (*n* = 3 fasted male mice for each compound).

^b^AUC, area under the plasma-concentration-time curve.

^c^*C_max_*, maximum plasma-concentration.

^d^*T_max_*_,_ time to reach the maximum plasma-concentration.

Dara are expressed as means ± SD.

### Inhibition of ACC reduces viral load in mice

Based on the safety, potency, and pharmacokinetics attributes of PF-05175157 in mice (Table 2) and humans [27], this compound was advanced for infection studies *in vivo*. For this purpose, WNV was selected as a model flavivirus because the murine model of WNV infection is more amenable and resembles clinical signs and pathology more accurately than DENV and ZIKV. Animals were treated with PF-05175157 (20 mg/kg) or drug vehicle twice a day p.o. and viral burden in serum and tissues was analyzed by quantitative RT–PCR up to 7 days after infection (Figure 2B). When compared to control animals, mice treated with PF-05175157 displayed a significant reduction of viral load in plasma at 3 and 4 days after infection (Figure 2C). Regarding the viral burden in other tissues, a significant reduction in the amount of viral RNA in the kidney and a tendency for a reduction in the amount of viral RNA in the lung from mice treated with PF-05175157 was noticed at 7 days post-infection (Figure 2D). Because PF-05175157 inhibits both ACC1 and ACC2 (Table 1), the usage of genetic models deficient on ACC1 or ACC2 was considered in order to dissect the role of ACC isoforms on flavivirus infection. Consequently, control wild type (WT) and ACC2 deficient (*ACC2*^-/-^) mice [31] were infected with WNV and the viral burden was compared (Figure 2E). Genetic depletion of ACC2 caused a significant reduction of the viral load at 4 days post-infection (Figure 2F). However, no significant differences were observed in the organs analyzed (brain, liver, kidney and lung) at 7 days after infection, although a tendency for a reduction in viral burden in the kidney of infected animals was observed (Figure 2G). These results suggested that the genetic depletion of ACC2 only partially reproduced the effect observed with PF-05175157, indicating that inhibition of ACC1 should be also necessary to achieve the effect observed with PF-05175157. Unfortunately, ACC1 deficiency is embryonically lethal, impairing the use of genetically engineered animal models to evaluate the role of this isoenzyme [33]. Overall, these results reveal the involvement of ACC in flavivirus replication in mouse models and prove the *in vivo* antiviral activity of ACC inhibitors such as PF-05175157.

### Mechanism of inhibition of flavivirus multiplication by ACC inhibitors

**Figure 3.**
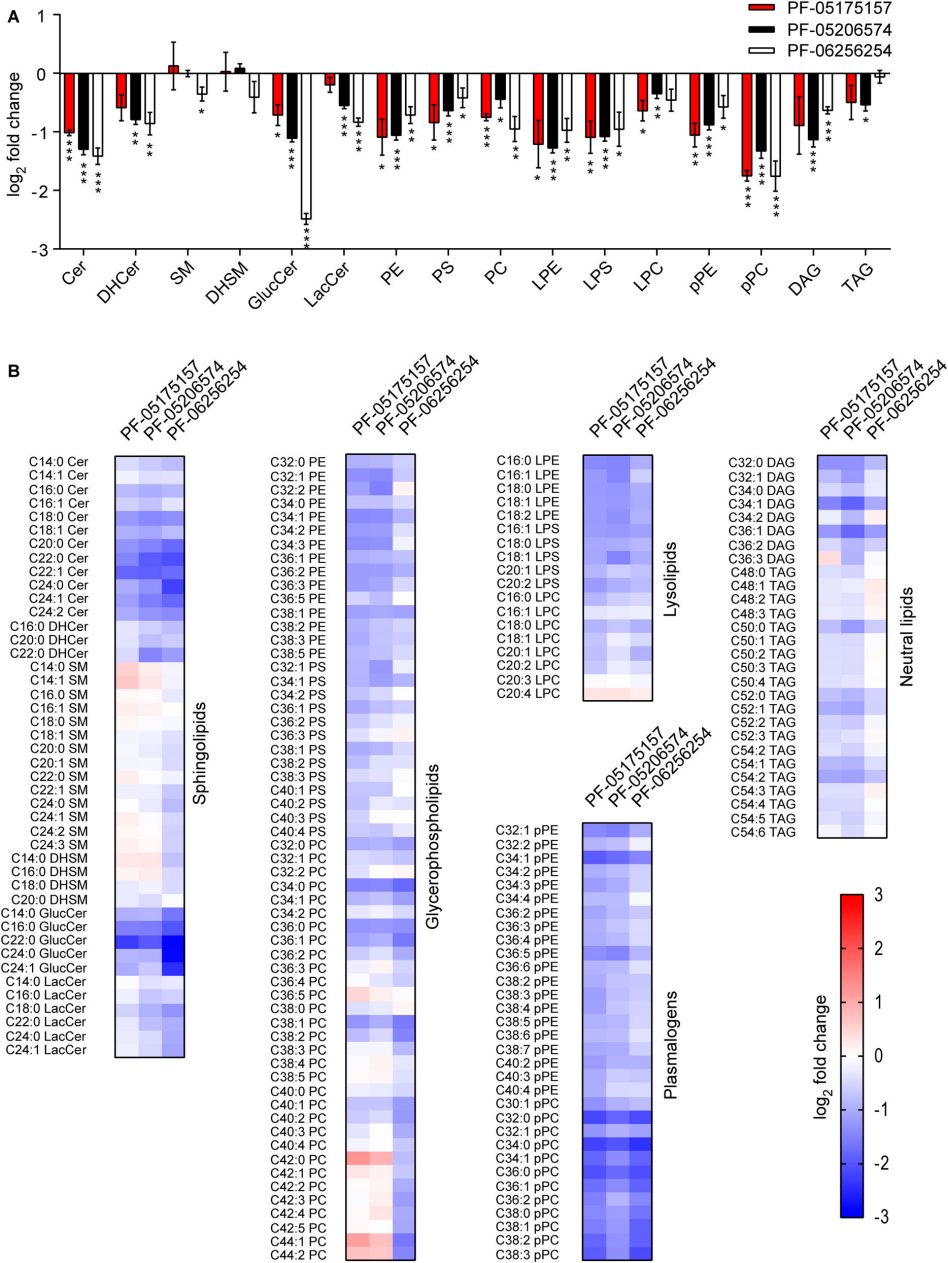
**Impact of ACC inhibitors on host lipids. (A)** Changes in the lipidome of Vero cells after 24 h of treatment with PF-05175157, PF-05206574 or PF-062562545 (5 µg/mL). Fold change in lipid levels were calculated as the log_2_ (Treated/Vehicle) for each lipid class. Data are expressed as the means ± SDs (*n *= 3 - 4). Two tailed Student’s *t*-test *P* values are Sidak-Bonferroni-corrected for multiple comparisons (*P <0.05, **P <0.01, ***P <0.0001). **(B)** Heatmap displaying the effect of PF-05175157, PF-05206574 or PF-06256254 on the individual lipid species analyzed. Cells were treated as described in A. Red and blue denote an increase or a decrease in lipid species, respectively. Data correspond to the mean of 3–4 repeats (*n* = 3-4). Sphingolipids (Cer, ceramide; SM, sphingomyelin), glycerophospholipids (PC, phosphatidylcholine; PE, phosphatidylethanolamine, and PS, phosphatidylserine), diacylglycerol (DAG), and triacylglycerol (TAG) were annotated using “C followed by the total fatty acyl chain length:total number of unsaturated bonds” and the “lipid subclass”. If the sphingoid base residue was dihydrosphingosine, the name contained a “DH” prefix. For glucosylceramides and lactosylceramides a “Gluc” or “Lac” prefix was respectively added to indicate the presence of the sugar moiety. Plasmalogens and lysophospholipids were annotated as described above, except that a “p” or “L” prefix was included, respectively.

**Figure 4.**
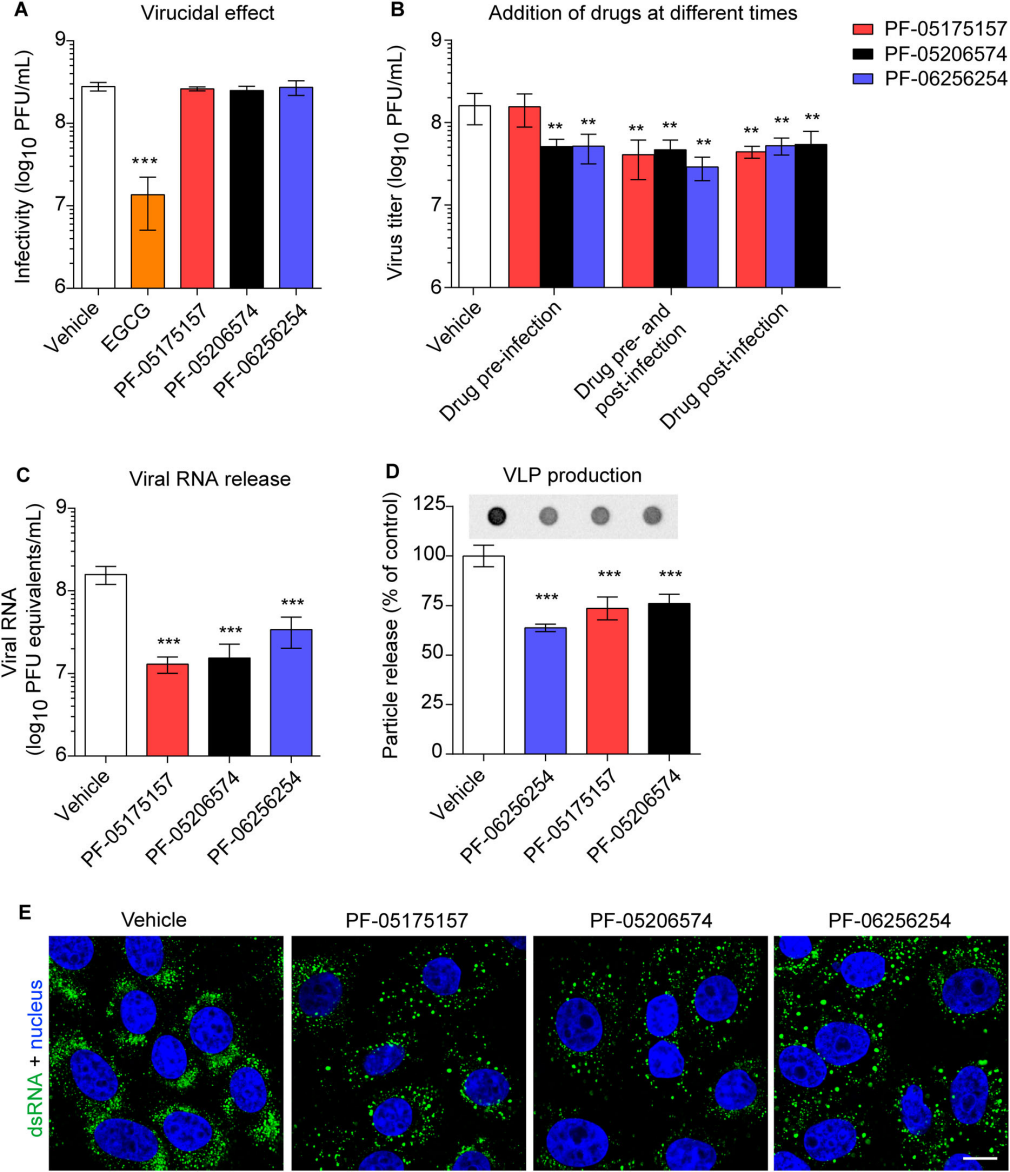
**Mechanism of inhibition of WNV multiplication by ACC inhibitors. (A)** Lack of virucidal effect of PF-05175157, PF-05206574 or PF-06256254. The same amount of WNV (∼ 3 × 10^8^ PFU) was treated with each compound (5 µg/mL) or drug vehicle for 1 h at 37°C in culture medium and the infectivity in each sample was determined by plaque assay. EGCG was included in the experiments as a positive control of a compound with virucidal activity (*n *= 3). **(B)** Time of addition experiments of ACC inhibitors. Vero cells were infected with WNV (MOI of 1 PFU/cell) and treated with PF-05175157, PF-05206574 or PF-06256254 (5 µg/mL) or drug vehicle only 1 h before virus inoculation (drug pre-infection), 1 h after virus inoculation (drug post-infection), 1 h before virus inoculation and during infection (drug pre-infection and post-infection), or left untreated (vehicle). Virus yield in supernatant was determined 24 h after infection by plaque assay (*n *= 3). **(C)** Determination by quantitative RT-PCR of genome containing particles released in cell cultures infected with WNV (MOI of 1 PFU/cell) and treated with ACC inhibitors (5 µg/mL) from 1 h after virus inoculation. Viral RNA was quantified 24 h after infection. **(D)** Production of WNV VLPs by HeLa cells stably transfected with a plasmid that expresses WNV prM and E proteins. HeLa3-WNV cells stably expressing WNV prM and E proteins were treated with ACC inhibitors (5 µg/mL) or drug vehicle and the amount of VLPs released to the culture medium was analyzed by enzyme-linked immunodot assay using a monoclonal antibody against WNV envelope protein as described in Material and methods. The graph displays the quantification of the amount of VLPs released (*n* = 4). **(E)** Detection of dsRNA in Vero cells infected with WNV (MOI of 1 PFU/cell) and treated with ACC inhibitors (5 µg/mL) from 1 h after virus inoculation. Cells were fixed and processed for immunofluorescence 24 h post-infection. Scale bar, 10 µm. In (A, B, C, and D) two tailed Student’s *t*-test *P* values between Vehicle and each compound and each treatment Bonferroni-corrected for multiple comparisons. *P < 0.05, **P < 0.01, ***P < 0.001. Data represent the means ± SDs.

The influence of ACC inhibitors on cellular lipids were investigated. For this end, lipidomics analyses of cells treated with the compounds were performed (Figure 3). A dose of the ACC inhibitors (5 µg/mL) that exhibited negligible cytotoxicity (Figure 1B) but significantly inhibited flavivirus replication (Figure 1C) was selected for these analyses. As expected, the ACC inhibitors significantly modified host lipid landscape, mainly decreasing a wide variety of lipids such as sphingolipids, glycerophospholipids, lysolipids, plasmalogens, and neutral lipids (Figure 3A and B). To identify the step of the flavivirus life cycle that was impaired by this alteration of lipid metabolism, WNV was selected again as a model. To exclude the possibility that the reduction of flavivirus production was caused as a result of a virucidal effect of the drug, the direct effect of the ACC inhibitors on the infectivity of WNV was evaluated. The same amount of plaque forming units (PFU) was preincubated with the compounds and then titrated to determine the remaining infectivity. In contrast to (-)-Epicgallocathechin gallate (EGCG), included as a positive control of a drug with virucidal effect [34], none of the ACC inhibitors assayed reduced WNV infectivity (Figure 4A). Consequently, time of addition experiments of ACC inhibitors showed that PF-05175157 did not affect WNV infection when added only prior to virus addition but reduced viral production when maintained after virus inoculation (Figure 4B). PF-05206574 and PF-06256254 reduced the infection when added prior to virus inoculation in a similar manner than when added after virus inoculation, suggesting subtle differences with PF-05175157 that could produce this long lasting effect. The ACC inhibitors significantly reduced the production of genome containing particles thus confirming the reduction in the production of progeny virions (Figure 4C). Flavivirus replication and morphogenesis are strictly connected to lipid metabolism [11]. To evaluate if the alteration of lipid metabolism was also related to an impairment of viral morphogenesis, a cell line that expresses WNV structural proteins and constitutively secretes virus like particles (VLPs) without the need of viral replication was used [32,35]. Immunoblot analysis of the amount of released VLPs indicated that the compounds significantly reduced the production of VLPs (Figure 4D). Flavivirus replication and morphogenesis take place coupled into specialized virus-induced membrane structures derived from the endoplasmic reticulum [36]. The visualization of flavivirus replication complexes by means of immunofluorescence detection of double-stranded RNA (dsRNA) intermediates confirmed that treatment with the ACC inhibitors had also an impact on the development of WNV replication complexes (Figure 4E). Taken together, these results suggest that the modification of lipid metabolism exerted by ACC inhibitors impaired flavivirus biogenesis by affecting both replication and particle morphogenesis.

## Discussion

The current progress in deciphering the puzzle of virus-host interactions is helping to develop alternative antiviral strategies. In this context, the close relation between flaviviruses and cellular lipids could drive to new therapeutic opportunities [11,37]. Although the recent increase on the information relative to the involvement of lipids in flavivirus infection [38–40], there are limited data supporting the role of host lipids during flavivirus infection *in vivo*, and only the relevance of cholesterol and sphingolipids has been demonstrated in animal models [41–44].

Taking advantage of a series of small-molecule ACC inhibitors recently developed to treat human disorders, we have investigated the significance of fatty acid metabolism in the infection of three different flaviviruses (WNV, DENV, and ZIKV) in cell culture. The studies were further extended *in vivo* using WNV as a model. The ACC inhibitors here used (PF-05175157, PF-05206574, and PF-06256254) displayed excellent *in vitro* and in cell culture properties (high potency and permeability, low cytotoxicity and good efficacy) and exhibited adequate pharmacokinetic parameters in animal models (like absorption or exposure). Our results showed that the ACC inhibitors reduced the multiplication of WNV, DENV, and ZIKV in cultured cells, which is compatible with the predicted broad spectrum of host targeting antivirals. Among the compounds tested, PF-05175157 has undergone multiple clinical trials in healthy volunteers (NCT01433380), and for the treatment of diabetes mellitus (NCT01792635). Consequently, PF-05175157 was selected as a lead compound for animal experiments. PF-05175157 reduced the peak of viremia in WNV-infected mice, supporting that ACC is a valuable target for antiviral intervention. This effect could be useful to reduce viral load, helping to control the infection or to diminish flavivirus transmission. In addition, PF-05175157 also reduced the viral burden in the kidney of infected mice. Importantly, WNV can induce persistent renal infection in humans and rodents causing chronic kidney disease [5,45], which means that the virus can be recovered from the urine of infected patients not only during acute infection, but also up to several years after infection [46–50]. Therefore, ACC inhibitors like PF-05175157 could turn into valuable tools for the prevention and treatment of renal complications associated to persistent WNV infection. Regarding the safety concerns for the therapeutic utilization of lipid metabolism inhibitors, the clinical success of the pharmacological manipulation of lipid metabolism with widely used drugs like statins or metformin has to be taken into consideration [51,52]. Moreover, metformin targets the adenosine monophosphate-activated protein (AMPK), which is a major regulator of the ACC activity, further supporting the safety of pharmacological manipulation of fatty acid metabolism as a potentially suitable therapeutic strategy [52]. Although treatment with ACC inhibitors failed to completely control viral infection, the reduction of viral load in serum and kidney of infected mice could contribute to the development of novel therapies to combat certain aspects of WNV infection.

The treatment with the ACC inhibitors elicited a major impact on cell lipid metabolism, as determined by lipidomics of treated cells. This effect was similar to the one described for another structurally-related ACC inhibitor [53]. A wide variety of lipids were reduced upon ACC inhibition. These included lipids with proven roles in flavivirus replication complex organization and morphogenesis like ceramides (Cer), glycerophospholipids, lysolipids, and neutral lipids [21,32,54–57]. Our results showed that treatment with ACC inhibitors had a direct impact on WNV infection by affecting both genome replication and particle assembly, two coupled processes strictly dependent on specific lipids necessary for membrane curvature, replication complex assembly, and virion envelopment [11,58]. Accordingly, the modulation of lipid fatty acid synthesis, impairs WNV multiplication [20,22]. The mechanism of inhibition of flavivirus multiplication of ACC inhibitors tested in this study was similar to the effect observed with other ACC inhibitors such as 5-(Tetradecyloxy)-2-furoic acid (TOFA) or 3,3,14,14-Tetramethylhexadecanedioic acid (MEDICA 16) [22]. Additionally, mouse experiments suggested that the inhibition of ACC1 (alone or together with ACC2) could be responsible for the antiviral effect of PF-05175157 in mouse models. This is consistent with the view that ACC1 has been mainly associated with lipogenesis, whereas ACC2 has been related to the regulation of fatty acid oxidation [24].

In summary, our results indicate that the manipulation of fatty acid metabolism via inhibition of ACC impairs flavivirus infection in cell culture and reduces WNV infection in animal models. Understanding the metabolic connections of viral infections opens new therapeutic opportunities using this type of inhibitors that could be ideally combined with other classical antiviral drugs such as nucleoside analogs to develop effective combinatorial therapies. Although these results provide a proof of concept, it has to be considered that further work directed to the optimization of dose and route is required to find the appropriate window of therapeutic efficacy. In addition, further chemical modifications of the compounds could contribute to maximize their antiviral effect.
